# An optimised protocol for detection of SARS-CoV-2 in stool

**DOI:** 10.1186/s12866-021-02297-w

**Published:** 2021-09-06

**Authors:** Tianqi Li, Enriqueta Garcia-Gutierrez, Daniel A. Yara, Jacob Scadden, Jade Davies, Chloe Hutchins, Alp Aydin, Justin O’Grady, Arjan Narbad, Stefano Romano, Lizbeth Sayavedra

**Affiliations:** 1 Gut Health and Microbes, Quadram Institute Bioscience, Norwich Research Park, Norwich, UK; 2 State Key Laboratory of Food Science and Technology, School of Food Science and Technology, Jiangnan University, Wuxi, China; 3 Microbes in the Food Chain, Quadram Institute Bioscience, Norwich Research Park, Norwich, UK

**Keywords:** FMT, COVID19, RT-qPCR, Stool, Clinical-test

## Abstract

**Background:**

SARS-CoV-2 has been detected in stool samples of COVID-19 patients, with potential implications for faecal-oral transmission. Compared to nasopharyngeal swab samples, the complexity of the stool matrix poses a challenge in the detection of the virus that has not yet been solved. However, robust and reliable methods are needed to estimate the prevalence and persistence of SARS-CoV-2 in the gut and to ensure the safety of microbiome-based procedures such as faecal microbiota transplant (FMT). The aim of this study was to establish a sensitive and reliable method for detecting SARS-CoV-2 in stool samples.

**Results:**

Stool samples from individuals free of SARS-CoV-2 were homogenised in saline buffer and spiked with a known titre of inactivated virus ranging from 50 to 750 viral particles per 100 mg stool. Viral particles were concentrated by ultrafiltration, RNA was extracted, and SARS-CoV-2 was detected via real-time reverse-transcription polymerase chain reaction (RT-qPCR) using the CDC primers and probes. The RNA extraction procedure we used allowed for the detection of SARS-CoV-2 via RT-qPCR in most of the stool samples tested. We could detect as few as 50 viral particles per 100 mg of stool. However, high variability was observed across samples at low viral titres. The primer set targeting the N1 region provided more reliable and precise results and for this primer set our method had a limit of detection of 1 viral particle per mg of stool.

**Conclusions:**

Here we describe a sensitive method for detecting SARS-CoV-2 in stool samples. This method can be used to establish the persistence of SARS-CoV-2 in stool and ensure the safety of clinical practices such as FMT.

**Supplementary Information:**

The online version contains supplementary material available at 10.1186/s12866-021-02297-w.

## Background

The global pandemic caused by SARS-CoV-2, poses an imminent threat to the global population. From December 2019 until the 22nd of June 2021, the number of confirmed cases stands at 179 million and rising, leading to an unprecedented challenge on health systems internationally. SARS-CoV-2 causes severe acute respiratory syndrome - infecting human cells by binding to the receptor angiotensin converting enzyme 2 (ACE2). ACE2 is an inflammation regulator expressed by epithelial cells located in the lung, liver, and gastrointestinal tract. It has been reported that gastrointestinal symptoms, such as diarrhoea, nausea, and vomiting, may be observed in up to 61 % of cases [1]. These gastrointestinal symptoms may be linked to the severity of the COVID-19 disease based on viral load and the degree of viral replication in the gut [2–5]. SARS-CoV-2 RNA has been detected in patient stool both during infection and after patients have apparently recovered – indicated by a lack of viral detection from nasal swab [6]. Viable SARS-CoV-2 has been isolated from stool samples [6–8], which suggests that there is a potential risk of faecal-oral transmission [9–13]. Hence, monitoring the viral load in stool is of crucial importance to maintain public health and limit viral spreading. A few studies have reported that the viral load in stool samples (10^2^-10^7^ genome copies mL^− 1^) is several orders of magnitude lower than in saliva (10^8^ genome copies mL^− 1^) [14, 15]. However, methods for the detection of the virus in stool have been poorly described. Robust and reliable methods are an urgent need, as microbiota-based therapies such as faecal microbiota transplantation (FMT) would need to rely heavily on the accurate screening of donor stools to ensure the absence of SARS-CoV-2 and guarantee patient safety [16].

For both nasal swabs and saliva samples, RT-qPCR is the most used diagnostic tool for detecting SARS-CoV-2, with many assays targeting the SARS-CoV-2 nucleocapsid (N) gene [17, 18]. For example, the commonly used Centers for Disease Control and Prevention (CDC) RT-qPCR test targets two regions of the N gene (N1 and N2). However, the faecal matrix has properties distinct from those of respiratory samples [19–21], therefore making the reliable detection of SARS-CoV-2 challenging. Recently, a few methods have been described for the detection of SARS-CoV-2 in stool samples [16, 22, 23]. However, the potentially low concentration of SARS-CoV-2 in faeces and the unique features of the sample matrix require optimised protocols to improve the recovery of viral RNA and increase our ability to detect the virus in stool samples. To address this need, we developed a reliable and sensitive method for SARS-CoV-2 detection in stool.

## Results

**Fig. 1:**
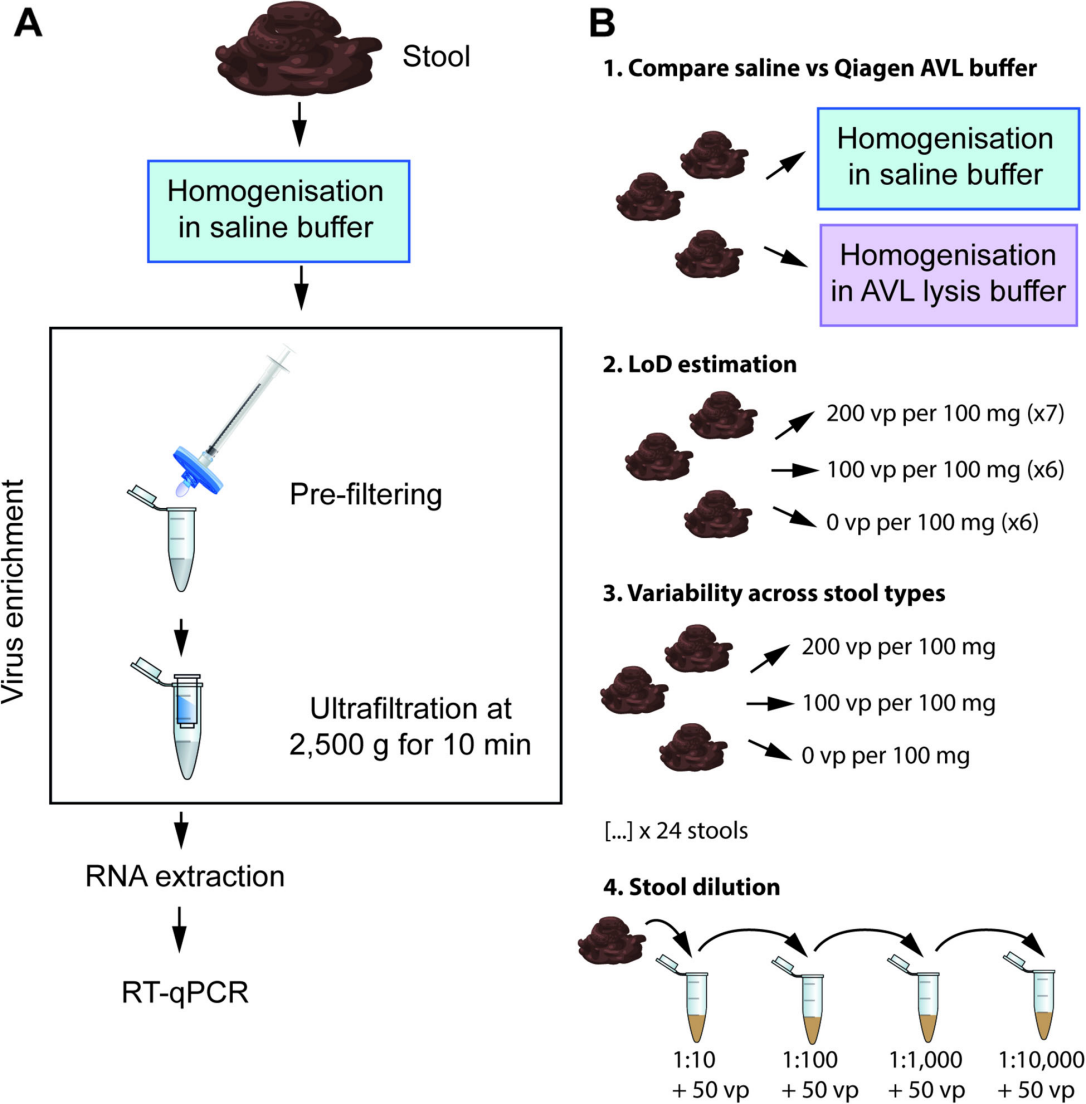
** A** Overview of our optimised method for SARS-CoV-2 detection in stool samples. **B** Schematic representation of the experiments conducted

Several recent studies indicate that viable SARS-CoV-2 can be detected in stool samples of COVID-19 patients [6, 7], suggesting that a possible risk for faecal-oral transmission exists. However, methods for the detection of SARS-CoV-2 in stool have been poorly assessed so far, and an optimised protocol is currently missing. Here, we describe an optimised protocol (Fig. 1A) to improve the detection of SARS-CoV-2 in stool samples. We performed our experiments using samples collected either before the current pandemic started (before October 2019) or from healthy donors who did not display and had not previously displayed symptoms of COVID-19. We used a commercially available SARS-CoV-2 stock, which was quantified through digital PCR by the manufacturer and was therefore used to infer the limit of detection of our approach. Extracted RNA samples were then used to detect SARS-CoV-2 via RT-qPCR with primer sets N1 and N2 [24]. We assessed how various steps throughout the RNA extraction influence the detection of SARS-CoV-2 in stool and report recommendations for optimizing these procedures in future clinical settings.

First, we spiked different volumes of an inactivated viral stock in stool samples and extracted the RNA with or without ultrafiltration (Supplementary Table 1). We obtained positive amplifications for both the N1 and N2 regions using both approaches. Without ultrafiltration we obtained positive amplifications with both N1 and N2 primer sets down to 2900 viral particles (vp) per 100 mg. In contrast, the addition of ultrafiltration allowed us to detect positive amplifications with both primer sets down to 725 vp per 100 mg (Supplementary Table 1). Hence, for all further tests we included an ultrafiltration step in our protocol. We then assessed what viral concentration was more reliably detected across stool types. Thus, we used a viral stock that had been accurately quantified using digital PCR and we spiked three stool samples using concentrations ranging from 50 to 750 vp per 100 mg. We were able to detect SARS-CoV-2 in all the stool samples we tested. The lowest concentration we could detect was 50 vp per 100 mg (Table 1), but a high variability amongst samples was observed for the lower concentrations (50–200 vp per 100 mg). This variability might be the result of the stool characteristics, for example, mucus and fibre content, as it has been previously reported that stool features can inhibit molecular assays [19–21].

**Table 1:** Ct values of RT-qPCR using RNA extracted from sample homogenised in saline buffer and AVL buffer

Viral load^a^	Saline buffer						AVL buffer					
	Stool 1		Stool 2		Stool 3		Stool 1		Stool 2		Stool 3	
	N1-mean	N2-mean	N1-mean	N2-mean	N1-mean	N2-mean	N1-mean	N2-mean	N1-mean	N2-mean	N1-mean	N2-mean
0	-	-	-	-	-	-	-	-	-	-	-	-
50	34.8	36.2	-	-	-	33.5	35.2	-	-	-		-
100	34.8	36.3	-	-	34.2	32.5	34.9	35.2	-	-	-	-
150	34.6	35.3	35.8	-	34.9	33.9	33.8	34.7	36.4	-	-	-
200	34.2	34.0	35.9	-	34.2	33.0	33.5	33.9	35.8	-	37.2	-
250	34.0	34.6	35.4	-	34.8	33.6	35.0	36.0	36.7	-	36.0	-
500	32.8	33.6	34.2	-	33.7	33.5	35.1	36.3	36.2	-	35.4	36.7
750	32.7	33.7	35.4	33.8	33.5	33.9	36.0	-	36.6	-	33.1	34.8

^a^ Numbers of viral particles spiked in 100 mg of stool

- = Sample showed no Ct value

We then selected the two lowest concentrations that gave reliable results (100 and 200 vp per 100 mg) to estimate the limit of detection (LoD) of our method. The stool sample used to determine the LoD was exclusively used for this experiment (i.e. was not used to create the previous datasets). We performed repeated extractions after spiking the homogenised stool sample with 100 vp per 100 mg (6 replicates in total) and 200 vp per 100 mg (7 replicates in total). As negative control, we also extracted 6 non-spiked stool aliquots, all of which resulted negative in the RT-qPCR assay. For the N1 primer set all samples spiked with either 100 or 200 vp per 100 mg resulted positive in the RT-qPCR assay (Table 2). In contrast, for the N2 primer set, 3 out of 6 and 7 out of 7 samples gave positive amplification for the 100 and 200 vp per 100 mg spikes, respectively (Table 2). Based on this data our approach has a LoD, defined as the lowest concentration at which all tested samples gave positive results in at least one RT-qPCR replicate, of 100 vp per 100 mg (1 vp mg^− 1^) for the N1 primer set and 200 vp per 100 mg (2 vp mg^− 1^) for the N2 primer set. These data are consistent with previous reports that highlighted the higher sensitivity of the N1 assay compared to the N2 assay [25].

**Table 2:** Overview of the limit of detected (LoD) experiments

	N1		N2	
Viral particles in 100 mg	100	200	100	200
Number of replicates	6	7	6	7
SARS-CoV-2 positive^a^	6 (100 %)	7 (100 %)	3 (50 %)	7 (100 %)
Mean Ct (st. dev)	32.6 (± 0.8)	31.4 (± 0.5)	32.8 (± 0.4)	32.1 (± 0.5)

^a^ Samples were considered positive if at least one RT-qPCR replicate showed Ct values < 40

**Fig. 2:**
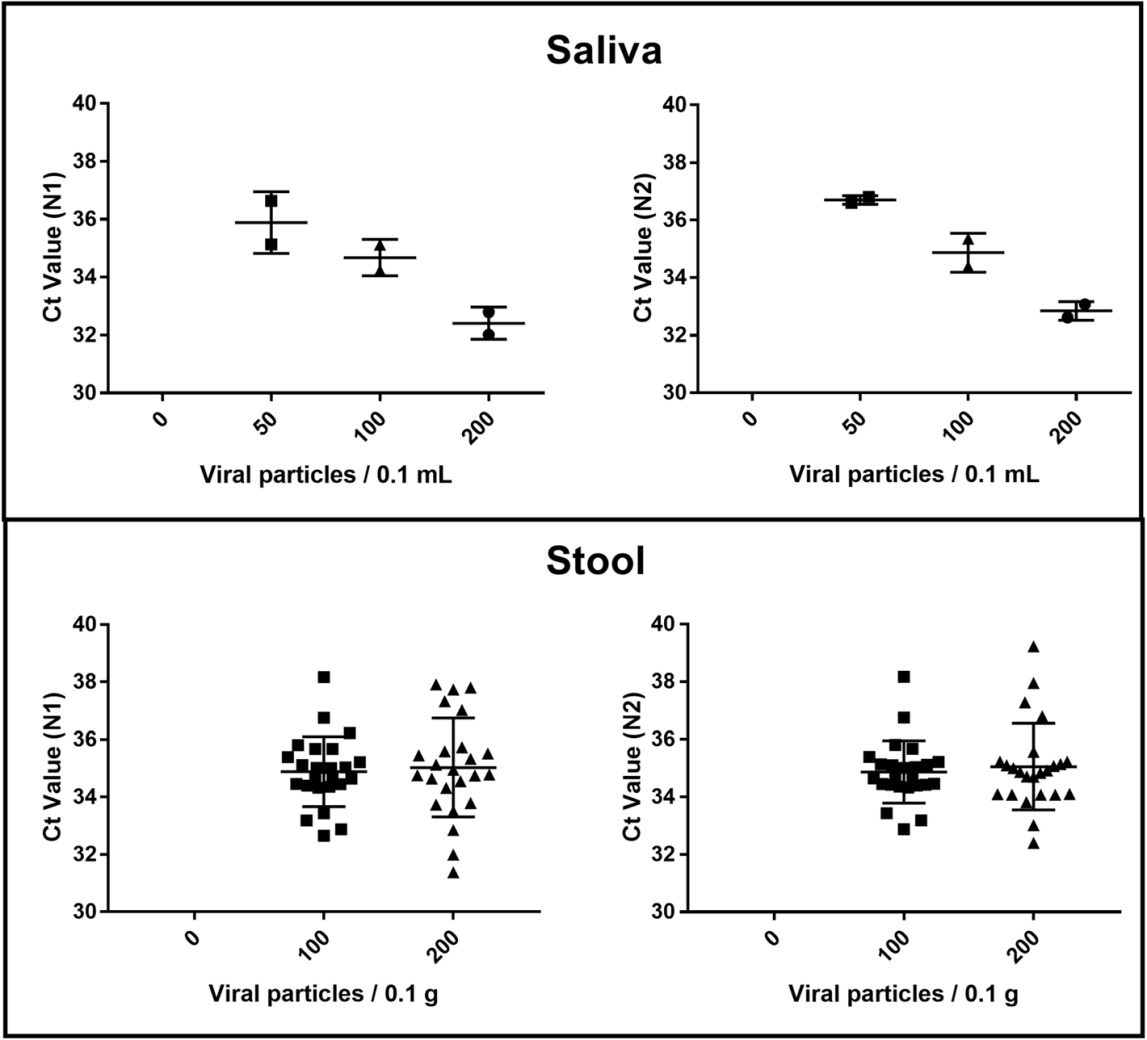
Ct values obtained for the N1 and N2 regions used to detect SARS-nCoV-2 in saliva or stool samples. The average of two technical qPCR replicates is shown. In five stool samples only one replicate gave positive results, which have been included in the graphs

Subsequently, we tested our method on 24 additional stool samples to estimate the variability in detection based on stool type and infer the specificity and sensitivity of our assay. We were able to detect the virus reproducibly and consistently across the majority of the samples with average Ct values ranging from 32.6 to 38.2 and from 32.0 to 37.7 for the N1 and N2 regions, respectively (Fig. 2). Using these 24 samples we estimated that our method has a sensitivity and a specificity of 100 % for both the N1 and N2 primer set (Table 3).

**Table 3:** Sensitivity and specificity of the protocol we developed. Data refer to the 24 stool samples spiked with 100 viral particles per 100 mg. The 95 % confidence interval is reported in brackets. A sample is considered positive if a single RT-qPCR replicate with Ct < 40 is detected for either the N1 or N2 primer set

Regions of N gene amplified	Sensitivity (*N* = 24)	Specificity (*N* = 24)
N1	24/24 (100 %, CI = 86–100)	24/24 (100 %, CI = 86–100)
N2	24/24 (100 %, CI = 86–100)	24/24 (100 %, CI = 86–100)

As we consistently detected viral particles in stool samples diluted in saline buffer, we investigated whether homogenising faecal material in the Qiagen AVL buffer would affect RNA recovery. For this test, we used the same three stool samples initially used to assess the lowest viral load detectable. When Qiagen AVL buffer was used, a higher variability was observed across samples (Table 1). Furthermore, we observed that the faecal material does not fully homogenise in the AVL buffer (Supplementary Fig. 1), suggesting that this might potentially affect the release of viral particles from the stool matrix.

Faecal material could potentially inhibit the PCR affecting the sensitivity and reproducibility of molecular assays. Hence, we performed serial dilutions of a selected stool sample, spiked them with the same amount of virus (50 vp per dulution), and processed them using our method. This was done to verify whether a decreasing amount of faecal material would affect Ct values. Higher sample dilutions did not decrease Ct values nor increased the consistency of positive qPCR results (Supplementary Fig. 2).

Finally, we applied our method to saliva samples (Fig. 2). We could consistently detect the spiked virus in all saliva samples tested. Moreover, we observed that doubling the vp concentration clearly resulted in a decrease of Ct values. This trend was not observed in the 24 stool samples we processed (Fig. 2). Hence, we recommend using our approach as a qualitative rather than quantitative method.

## Discussion

Initial reports indicate that the concentration of SARS-CoV-2 in stool might be several orders of magnitude lower than in saliva [14, 15]. Moreover, the complexity of the stool matrix can affect the precision of molecular testing [19–21], posing additional challenges for the detection of the virus in stool samples. Hence, methods that can account for these limitations are needed to allow a robust and reliable detection of the virus in faeces. By concentrating the viral particles via ultrafiltration, we were able to develop a sensitive method to detect SARS-CoV-2 in faeces, with a LoD of 1000 vp g^− 1^ for N1 and 2000 vp g^− 1^ for N2. To the best of our knowledge, this is the lowest LoD so far described [16, 26]. Manzoor et al. reported a LoD of 204 vp g^− 1^ [27]. However, this was calculated as the lowest copy number that could be detected in stool spiked with extracted RNA, without considering the reproducibility of this measure across multiple replicates as we have reported in our study.

The method we describe has high sensitivity and specificity. It is worth noting that roughly half of the initial faecal slurry was used after centrifugation (500–600 µL of the 1 mL in which 100 mg stool was homogenised). Hence, if we assume a homogeneous suspension, the Ct values we obtained are from roughly half the initial number of viral particles spiked. Moreover, following the recommendation in the RNA extraction kit manufacture’s protocol we eluted the extract twice with 40 µL buffer. Although the elution with this volume (2 × 40 µL) might increase the total yield of RNA, an elution with lower volume could result in a more concentrated extract and therefore, increase the sensitivity of the RT-qPCR assay. Altogether these data indicate that, potentially, our method has room for further improvements by enhancing the separation between debris and supernatant to recover higher fractions of the slurry used in the RNA extraction and by using smaller volumes for RNA elution.

We report that diluting the stool samples in AVL leads to less accurate results. This observation is particularly relevant because in several studies, RNA has been extracted from stool samples by applying the standard manufacturers’ protocol of the extraction kits [13, 28]. Such protocols recommend dissolving samples in extraction buffers (e.g. Qiagen AVL buffer), and are not necessarily optimised for stool samples. Our data indicate that depending on the stool matrix type, this procedure can reduce the efficiency of the RNA extraction and possibly underestimate the virus detection. Hence, we strongly recommend following optimised protocol for stool, like the one we discuss here, that require the dissolution of the sample in saline solution before RNA extraction.

## Conclusions

Here we assess the technical challenges encountered while screening stool material for the presence of SARS-CoV-2. We describe a robust approach to detect SARS-CoV-2 in stool samples, having a LoD of 1 and 2 vp mg^− 1^ for the N1 and N2 primer set, respectively. Although we could detect as low as 50 vp per 100 mg, a high variability can be observed between stool sample types when viral concentrations are low. We demonstrated that following standard manufacturer’s RNA extraction protocols may not be sufficient to detect SARS-CoV-2 in stool samples as stool consistency and homogenisation media can affect the downstream assays. To improve detection, we strongly recommend homogenising the stool samples in saline solution first, then concentrating the viral particles with ultrafiltration. Our method is sufficiently reliable to monitor the prevalence and persistence of the viral particles in the gut and can help in determining the safety of samples intended for use in FMT applications. To ensure the maximal safety for the patients, we propose that FMT donors should be screened following the recommendations here, and donor stool should be excluded even if a single N region (N1 or N2) or a single replicate per region gives a positive result due to the variability introduced by the stool matrix.

## Method

### Sample preparation and RNA extraction

Stool samples collected either before the COVID-19 outbreak (October 2019), or from donors who did not display and had not displayed symptoms of COVID-19 were weighed (100 mg) and homogenised in saline solution (0.89 % w:v NaCl) with a ratio of 1:10 (w:v; 100 mg in 900 µL) by vortexing for at least 1 min. In an initial screening phase, we assessed the effect of an ultrafiltration step aimed at enriching viral particles before RNA extraction using a single stool sample. For these tests, we spiked the homogenised stool with different volumes of AMPLIRUN® TOTAL SARS-CoV-2 CONTROL (VirCell Microbiologists, Spain) and then either directly extracted the RNA using the QIAamp Viral RNA Mini Kit (Qiagen, UK; CN 52,906) or, first enriched the viral particles by ultrafiltration and then extracted the RNA, as described below. Since the ultrafiltration step improved SARS-CoV-2 detection we added this step in all further RNA extractions.

All the following experiments were performed by spiking the homogenised stool samples with the inactivated stock solution of SARS-CoV-2 positive Q control, SCV2QC01-B, Qnostics (UK). We used this stock because it has been precisely quantified by the manufacturing company using digital PCR. The stock is available by the supplier (Randox Biosciences, UK) in transport media at a viral concentration of 10,000 digital copies (dC) mL^− 1^. The homogenised stool samples (1 mL) were spiked with different concentrations of viral particles, centrifuged at 4,000 g for 10 min, and then supernatants were filtered through 0.22 µM syringe filters. Virus enrichments were then performed using ultrafiltration tubes (Sigma, UK; CN UFC810024) by loading 500 µL of the filtrate and centrifuging at 2,500 g for 10 min. The concentrated samples (around 50 µL) were used for RNA extraction using QIAamp Viral RNA Mini Kit, following the protocol provided by the manufacturer. Finally, the viral RNA was eluted in two aliquots of 40 µL buffer AVE supplied with the kit as recommended by the manufacturer to increase the yield. An overview of the method we developed is reported in Fig. 1A. We first used three stool samples to assess the lowest amount of virus detectable with our approach. The same samples were then used to assess the effect of the solution used to homogenise the stool (Table 1). This was done by repeating the above protocol and homogenising the stool not in saline solution but in AVL Buffer (Qiagen, UK; CN 19,089), as recommended in the QIAamp Viral RNA Mini Kit manufacturer manual. Finally, 24 additional stool samples were then used to verify the variability of the assay across stool types and to infer the sensitivity and specificity of our protocol by extracting RNA after spiking them with 0, 100 and 200 vp per 100 mg.

An additional stool sample was then used to calculate the limit of detection (LoD) of our method. The LoD, defined as the lowest concentration of virus at which all samples had at least one positive RT-qPCR replicate, was estimated as follows. A freshly collected stool sample was diluted in saline solution in the ratio specified above and then stored at -20^o^ C until further processing. After thawing, the homogenised stool sample was aliquoted and six and seven replicates were spiked with 100 or 200 vp per 100 mg, respectively. Six non-spiked replicates were also prepared. Samples were processed as described, and virus enrichment was performed using between 500 and 600 µL of supernatant. RNA was extracted as described. To verify the absence of contamination in the reagents, we also performed RNA extractions using kit reagents only. As positive control, we extracted RNA from 10 µL of the original stock used to spike the samples.

Finally, we selected one stool sample and performed serial dilution to verify whether decreasing the level of faecal material could improve the qPCR results. Here, 100 mg of stool was homogenised in 900 µL of saline solution. Subsequently, the homogenate was diluted down to 1:10,000 in 10-fold dilution steps. All dilutions were spiked to a final concentration of 50 vp/sample. Samples were processed for RNA extraction and qPCR as described above. An overview of the experiments performed in this study is reported in Fig. 1B.

We then tested our method on two saliva samples that were collected before the start of the COVID-19 pandemic. Here, 100 µL of saliva was diluted in 900 µL of saline solution and were then spiked with the inactivated SARS-CoV-2 viral stock (SCV2QC01-B) with a final concentration of 0, 50, 100 or 200 vp per 100 µL of saliva. The samples were then processed using the same procedure described for stool.

### RT-qPCR assay

Primer sets N1 and N2 (Integrated DNA Technologies, Belgium, 10,006,713) were used for identifying SARS-CoV-2 with the Probe 1-Step Go No Rox or Probe 1-Step Go Rox kits (PCR Biosystems) [24]. RT-qPCR was then performed in a Roche LightCycler® 480 Instrument II or a StepOnePlus™ Real-Time PCR System (Applied Biosystems™) using the following conditions: 50ºC for 10 min, 95ºC for 2 min, 45 cycles of 95ºC for 5 s, 55ºC for 30 s, followed by 40ºC for 30 s. The Ct values were calculated, and samples were considered as positive only if they showed Cts lower than 40 cycles. RT-qPCR was always performed using two technical replicates. As the aim of this study was to verify the variability in detecting SARS-CoV-2 in stool samples with the intention of reporting robust guidelines for screening FMT material, we considered positive also samples for which only 1 RT-qPCR technical replicate gave positive amplification. To determine the 95 % confidence interval for the specificity and the sensitivity we used the *binom.test* function in the R software [29].

## Supplementary Information

**Additional file 1.**

## Data Availability

Data sharing is not applicable to this article as no datasets were generated or analysed during the current study.

## Abbreviations

FMT: Faecal microbiota transplant
vp: Viral particles
LoD: Limit of detection
