# Supplementary material for: An optimised protocol for detection of SARS-CoV-2 in stool
Source: BMC Microbiol. 2021 Sep 6;21:242. doi: 10.1186/s12866-021-02297-w (PMC8419809; doi:10.1186/s12866-021-02297-w)
Supplement: Supplementary file 1 — Additional file 1. [file 12866_2021_2297_MOESM1_ESM.docx]

Supplementary materials


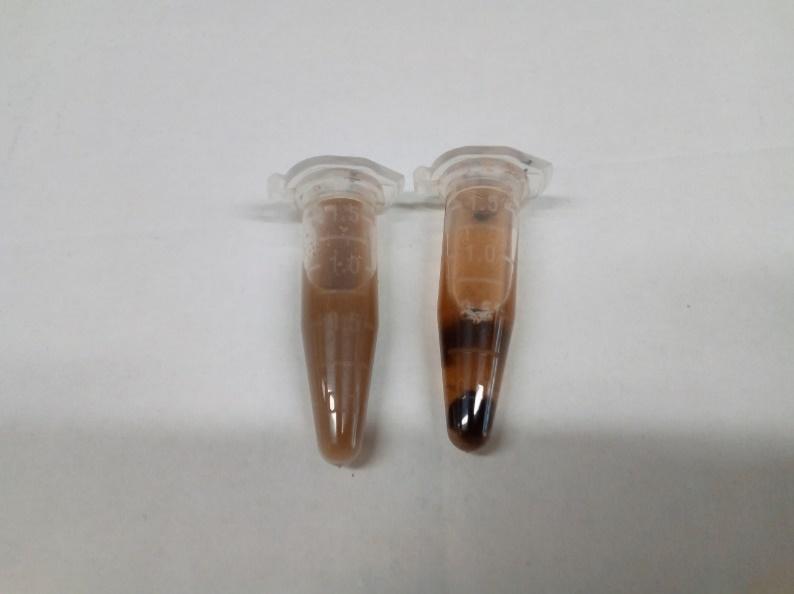


Supplementary Figure 1. Faecal samples mixed and resuspended in 1 ml of saline buffer (left) resulted in a better homogenisation of the stool as compared to using AVL buffer (right).


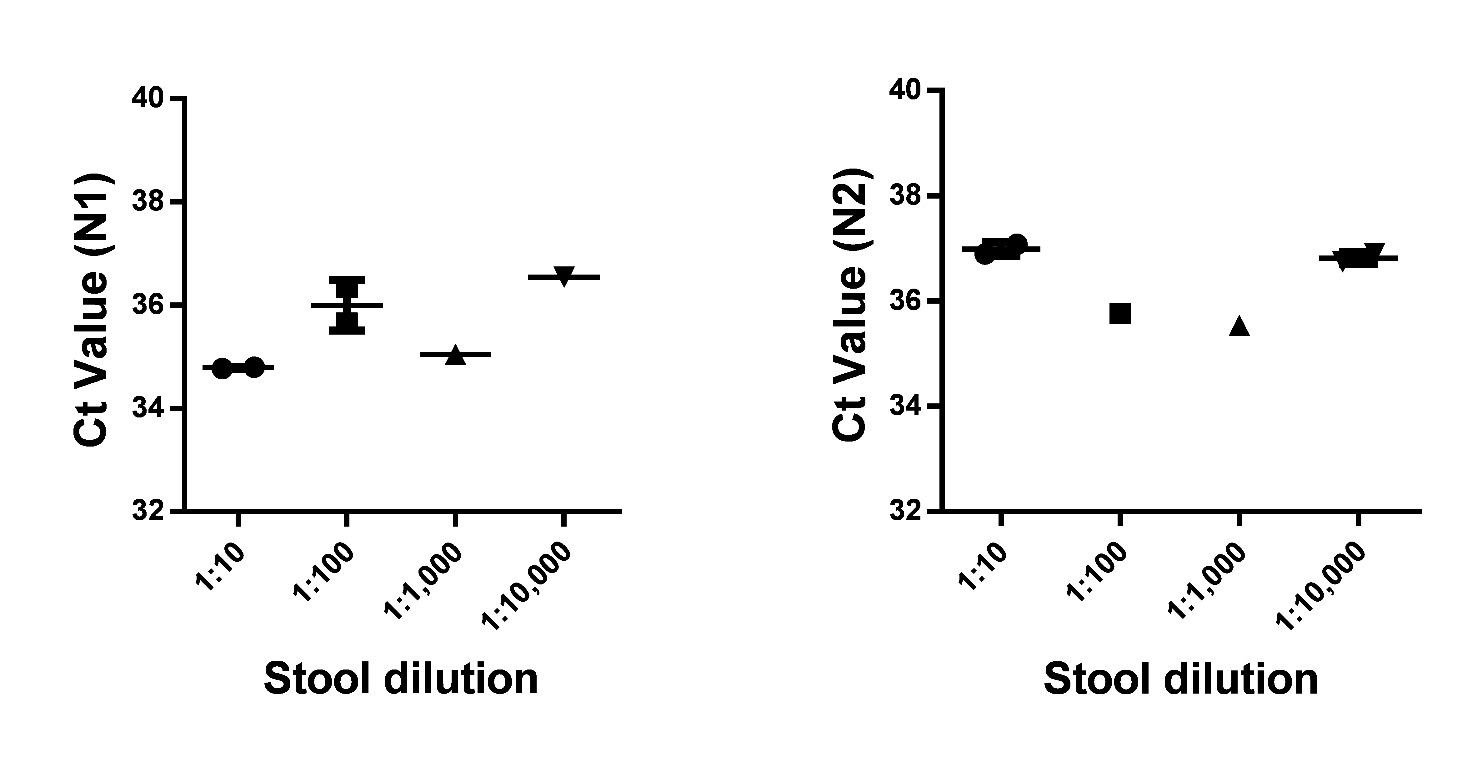


Supplementary Figure 2. CT values obtained with serial dilutions of stool using the N1 or N2 primer sets.

Supplementary Table 1. Ct values obtained from stool samples spiked with different amounts of viral particles used to extract RNA with or without ultrafiltration.

|  | Without ultrafiltration | | | | With ultrafiltration | | | |
| --- | --- | --- | --- | --- | --- | --- | --- | --- |
| Spiked viral particles (vp in 100 mg) | N1 | N1 | N2 | N2 | N1 | N1 | N2 | N2 |
| 2900 | 32.3 | 31.9 | 31.4 | 31.3 | nt | nt | nt | nt |
| 1450 | 32.6 | 33.6 | - | - | 32.7 | 32.6 | 31.3 | 31.6 |
| 725 | nt | nt | nt | nt | 33.2 | 32.5 | 33.5 | 33.6 |

nt = not tested

- = Sample showed no Ct value
